# Research on the influence mechanism of social risk perception on addictive behavior among sports lottery gamblers: the moderating effect of social support

**DOI:** 10.3389/fpsyg.2025.1704105

**Published:** 2025-12-22

**Authors:** Menglong Lin, Shunfang Liu, Feng Wang

**Affiliations:** 1 Faculty of Sport and Leisure, Guangdong Ocean University, Zhanjiang, China; 2 School of Physical Education and Sports Science, South China Normal University, Guangzhou, China

**Keywords:** behavioural addiction, lottery gamblers, moderating effect, perceived social risk, social support

## Abstract

**Introduction:**

Against the backdrop of rapid social transformation, emerging social issues have heightened public uncertainty and risk perception. In this context, the consumption of China's sports lottery has expanded rapidly. While this growth benefits sports development and public welfare funding, it has also raised concerns about gambling addiction and associated psychosocial problems. Grounded in social cognitive theory, this study investigates the mechanism through which social risk perception influences sports lottery gambling addiction, and examines the moderating role of social support. The aim is to provide theoretical insights for policy interventions and the promotion of responsible gambling behavior.

**Methods:**

A survey was conducted with 362 Chinese sports lottery consumers. Data validity and reliability were assessed using SPSS 25.0. Correlation and multiple regression analyses were performed to test the relationships among the variables.

**Results:**

Results indicate that social risk perception significantly and positively predicts sports lottery gambling addiction. Moreover, social support significantly moderates this relationship, such that higher levels of social support weaken the positive impact of social risk perception on addictive behaviors.

**Discussion:**

The findings highlight the complex role of psychosocial factors in addictive behaviors and provide a theoretical basis for targeted interventions. Strengthening social support networks and improving policy design may reduce social risk perception, thereby preventing and mitigating gambling addiction among lottery players. These results underscore the practical value of social support in public health policy.

## 1 Introduction

Against the backdrop of rapid social change and ongoing modernization, new types of social problems continue to emerge, accompanied by a marked increase in individuals' perceptions of social uncertainty and risk. Intensifying social insecurity, feelings of isolation, material losses, and economic pressures have become key factors affecting public mental health and disrupting the stability of everyday life. This phenomenon reveals that, in modern society, the relationship between individuals and social risks is increasingly intertwined, placing everyone in an environment of unavoidable risk coexistence. As previous studies have noted, individuals' subjective perceptions of social adversity are significantly associated with heightened risks of suicide and maladaptive behaviors, such as gambling and substance abuse (刘慧瀛, 郝祥森, 2022).

In this context, the scale of China's sports lottery consumption has continued to expand. On the one hand, this growth supports the development of sports and public welfare; on the other hand, it has drawn increasing attention to gambling addiction and its potential psychosocial consequences. According to general strain theory (Agnew and White, 1992), when individuals encounter external stressors or socially tense situations, they often experience negative emotions such as anxiety, depression, and fear, and are more likely to engage in maladaptive behaviors (e.g., addictive gambling) to alleviate psychological distress. 石永东 and 蒲小红 (2017) further emphasized that negative emotions themselves are important triggers of gambling addiction. This suggests that in the face of increasing social risks and economic pressures, individuals may engage in compulsive lottery gambling as a means of avoiding or displacing their perceived sense of social threat.

In addition to its rapid expansion, sports lottery gambling has raised growing concerns regarding its potential clinical and societal impacts. Globally, problem gambling has been recognized as a major public-health issue, with recent meta-analytic evidence showing that approximately 2.3% of adults meet the criteria for problem gambling, and an even greater proportion experience subclinical gambling-related harm (Calado and Griffiths, 2016). Sports betting and lottery gambling—often perceived as “low-risk” or “socially acceptable” forms—have nevertheless been identified as significant contributors to gambling-related problems, including financial strain, impaired social functioning, and increased psychological distress (Sarti and Triventi, 2017). Epidemiological data also indicate that gambling disorder is strongly associated with depression, anxiety, impulsivity, and elevated risks of suicidal ideation and attempts (Armoon et al., 2025). In China, although large-scale national epidemiological surveys remain limited, existing evidence suggests that the rapid growth of lottery participation coincides with heightened vulnerability among certain subgroups, especially those experiencing economic pressure or social instability. Therefore, examining the psychological mechanisms underlying sports lottery gambling addiction is not only theoretically relevant but also crucial for informing public-health interventions in the Chinese context.

Notably, SS, as a critical social resource, is widely recognized for its buffering and moderating effects on the influence of external stressors on individual behavior. Empirical studies have shown that SS can effectively mitigate the disruptive impact of behavioral addictions—such as internet addiction, mobile phone addiction, and SNS addiction—on individuals' daily lives and interpersonal relationships (Johnson and Jennison, 1994; Marshal and Chassin, 2000). This suggests that when analyzing how social risk perception (SRP) influences sports lottery gambling addiction, SS may function as a moderator and serve as an important psychological mechanism shaping individuals' gambling behavior.

Social Cognitive Theory (SCT), proposed by Bandura, provides an essential theoretical foundation for understanding how individuals interpret their environment, regulate their emotions, and decide whether to engage in certain behaviors. SCT emphasizes the reciprocal interaction among personal cognitive factors (e.g., beliefs, expectations, self-efficacy), environmental influences, and behavioral outcomes (Bandura, 1986, 2014). According to SCT, individuals' perceptions of external events are filtered through cognitive appraisals, which shape their emotional responses and ultimately guide behavioral tendencies such as risk-taking or avoidance.

Within the context of gambling behaviors, SCT suggests that subjective perceptions—such as perceived social risk, anticipated emotional relief, and beliefs about gambling outcomes—play a central role in explaining why individuals may choose gambling as a coping strategy. Individuals who perceive high levels of social risk may form negative cognitive appraisals (e.g., uncertainty, insecurity), which in turn amplify maladaptive coping tendencies and increase the likelihood of engaging in addictive gambling behaviors (Hurel et al., 2019). At the same time, SS may influence this cognitive–behavioral process by reshaping emotional evaluations, providing alternative coping resources, and reducing the perceived necessity of gambling as an emotional escape. Through this lens, SCT not only clarifies how SRP contributes to gambling addiction but also explains why SS may moderate this relationship by functioning as a cognitive and emotional buffer (van der Maas, 2016).

Based on SCT, the variables and hypotheses in the present study were operationalized to capture the cognitive mechanisms linking environmental stressors (SRP) to maladaptive behavioral responses (sports lottery gambling addiction), as well as the moderating influence of a key social–cognitive resource (SS). This theoretical grounding ensures that the hypothesized pathways reflect the SCT principle that cognitive processes mediate and regulate behavioral outcomes in stressful social contexts.

Based on the above perspectives, this study focuses on sports lottery gamblers (SLGs) as the target population, aiming to systematically examine the mechanisms through which SRP influences gambling addiction, and to further test the moderating role of SS. By developing a theoretical model and conducting empirical analyses, the study seeks to uncover the complex relationships among structural pressures, subjective perceptions, and maladaptive behaviors. The findings are expected to provide a theoretical basis and practical guidance for responsible lottery consumption, risk intervention, and the promotion of mental health in China.

## 2 Literature review

### 2.1 The relationship between SRP and gambling addiction in sports lottery

Within the highly complex and dynamic structure of contemporary society, individuals' subjective perceptions of social risk have intensified. Such perceptions manifest not only in uncertainty about the future but also in persistent experiences of psychological stress and anxiety in daily life. Prior research indicates that anxiety stemming from social risk may not merely represent short-term emotional fluctuations but can develop into chronic and pathological psychological distress, such as prolonged depression, low mood, helplessness, or even suicidal ideation (Yeom and Choi, 2014). Such emotional difficulties are often closely associated with negative cognitions about the future and are highly comorbid, particularly pronounced among individuals with co-occurring depressive and anxiety disorders (Yoon and Jang, 2019).

Building on this psychological mechanism, scholars have found that gambling behavior is often regarded as a strategy for coping with or escaping negative emotions, and is significantly associated with symptoms of anxiety and depression. McCormick et al. (1984) reported that individuals with high levels of depression and low self-esteem prior to developing gambling addiction were more likely to engage in gambling and experienced temporary psychological relief during the activity. Particularly outside gambling episodes, gamblers often exhibit heightened depressive symptoms. This suggests that gambling may function as a means of escaping social stress or emotional distress rather than serving merely as a recreational activity.

Furthermore, Perkins (1999) suggested that chronic and intense social stress may elicit persistent negative emotions such as tension and anxiety, and significantly increase individuals' reliance on substitute behaviors, including gambling and internet addiction. Jacobs (1986) also noted that the sense of “immersion” or “dissociation” experienced under stressful conditions can temporarily reduce real-world anxiety, allowing individuals to momentarily “escape” distressing realities and thereby facilitating the onset of addictive behaviors. In other words, when confronted with uncontrollable or unpredictable social risks, individuals are more likely to adopt maladaptive, addictive behaviors—such as excessive sports lottery gambling—as a means of alleviating stress and anxiety.

A synthesis of existing studies suggests that SRP, as a subjective experience rooted in structural tensions, is closely linked to maladaptive behaviors such as gambling addiction. In the context of increasingly prevalent gambling behaviors, clarifying the pathways through which SRP affects gambling addiction holds considerable theoretical and practical significance. However, systematic investigations of this mechanism remain limited, particularly among SLGs, where the interplay of relevant variables has yet to be thoroughly examined.

### 2.2 The relationship between SRP and SS

In social psychology research, although relatively few studies have directly examined the relationship between SRP and SS, numerous studies have highlighted the critical role of SS in coping with stress and mitigating risk. 王刚 and 宋锴业 (2018) found a significant negative correlation between government support and individuals' perceptions of social risk, suggesting that SS can partly alleviate individuals' subjective perceptions of social risk. Specifically, individuals with higher levels of SS are able to draw upon multiple resources from society, family, or organizations when facing uncertain or threatening events, thereby reducing feelings of isolation and helplessness and alleviating excessive concerns about potential threats. In contrast, individuals lacking effective SS are more likely to experience intense fear, anxiety, and helplessness when dealing with social risks, which in turn reinforces their perception of risk uncontrollability.

SS is commonly defined as the emotional, informational, and material resources that individuals obtain through social relationships, which enhance their perception of being loved, protected, and supported (Barrera, 1986). Bolger et al. (2000) further emphasized that when individuals experience stress or adversity, SS not only provides emotional comfort but also substantially strengthens coping capacity. Consequently, SS has been regarded as an essential “psychological buffer” that mitigates the adverse effects of catastrophic events (Norris et al., 2008).

Regarding the mechanisms of SS, scholars have primarily explained them from two theoretical perspectives. The first is the direct effect theory, which posits that SS exerts a positive influence by providing stable resources in everyday life, thereby exerting its benefits even before stressors occur. For example, access to social information, advice, or resources can help prevent the occurrence of certain social risks (Overholser et al., 1990). The second is the stress-buffering theory, which emphasizes that SS alleviates the negative emotional and behavioral consequences of stress through emotional comfort and resource regulation, thereby restoring psychological balance and enhancing coping capacity (An, 2016).

Moreover, research on post-disaster recovery has further validated the beneficial role of SS in promoting mental health. It not only serves as a vital force in helping individuals recover from crises but also enhances cognitive perceptions of safety and controllability in the environment. Therefore, it can be inferred that in the process by which SRP influences behavioral decision-making, SS functions not only as an objective resource but also as a cognitive cue, exerting a critical influence on emotional regulation and behavioral choices.

In summary, SS may act as a moderating factor in the relationship between SRP and maladaptive behaviors. For SLGs with heightened levels of SRP, SS may attenuate gambling addiction tendencies through mechanisms of emotional buffering and behavioral guidance. Thus, further exploration of the moderating effect of SS in the “SRP–gambling addiction” pathway will help elucidate the psychological dynamics underlying this mechanism and provide a theoretical foundation for behavioral intervention.

### 2.3 The relationship between SS and gambling addiction

Existing research demonstrates that SS plays a critical role in the intervention and buffering mechanisms of behavioral addictions, particularly gambling addiction. Overall, lower levels of SS are frequently associated with a higher risk of behavioral addiction, as individuals become more vulnerable to external pressures and are more likely to engage in maladaptive behaviors. Conversely, higher levels of SS can effectively reduce the likelihood of gambling addiction and other psychosocial problems (Yoon and Park, 2018). This indicates that SS is not only an emotional resource but also a vital social protective factor against behavioral addictions.

In research on adolescent internet addiction, Kim et al. (2016) found that SS exerts a significant moderating effect: adolescents with higher levels of SS exhibited significantly lower levels of internet addiction compared to those with lower levels of support. Similarly, Jung (2024), in examining the effects of anxiety, depression, and impulsivity on adolescents' smartphone addiction, confirmed that SS has a significant buffering and moderating function in this relationship. Taken together, these studies suggest that SS not only reduces the likelihood of behavioral addictions but also weakens individuals' reliance on addictive behaviors when confronted with negative emotions.

In the field of gambling addiction research, SS has been widely recognized as a critical protective factor. It not only helps reduce the incidence of gambling addiction but has also been incorporated into intervention and treatment programs (Gomes and Pascual-Leone, 2009; Petry and Weiss, 2009). These studies suggest that the prevention and treatment of gambling addiction rely not only on individual willpower but also on the social networks and environmental factors in which individuals are embedded. A strong SS system can enhance individuals' self-regulation, strengthen their psychological resilience against external pressures and temptations, and thereby reduce their dependence on gambling behaviors.

In summary, previous research has widely acknowledged the mitigating effects of SS on various behavioral addictions, highlighting its role in providing emotional relief and behavioral guidance when individuals face high-pressure situations. Building on this evidence, the present study incorporates SS as a moderating variable to further examine its role in the relationship between SRP and sports lottery gambling addiction. By elucidating this psychological mechanism, the study aims to provide empirically grounded theoretical support for behavioral interventions and the promotion of responsible lottery gambling.

## 3 Methods

### 3.1 Survey participants

This study focused on SLGs as the target population, aiming to analyze the impact of SRP on gambling addiction and to further test the moderating role of SS in this relationship. To ensure relevance and representativeness, individuals with prior experience in purchasing sports lottery tickets were selected as the sample source.

Data were collected through a combination of online and offline questionnaires. The purpose of the study and detailed instructions were provided at the beginning of the questionnaire. To improve the scientific validity and diversity of the sample, stratified random sampling was employed. A stratified random sampling procedure was applied to improve representativeness. Stratification was based on two variables: gender (male/female) and age group (18–29, 30–39, ≥40), reflecting the demographic composition of the SLG population reported in national market statistics. These strata were chosen because gambling behavior is known to vary by gender and age, and proportional allocation was applied so that the sample distribution matched the estimated distribution of the target population. Within each stratum, participants were randomly selected through online and offline recruitment channels. Prior to the formal distribution, a small-scale pilot test was conducted to optimize the questionnaire structure and wording. During the formal survey, questionnaires were distributed via online platforms (e.g., Wenjuanxing, social media groups) and selected offline channels. Given the large number of questions and the time required to complete them, participants were offered modest incentives to encourage full participation.

A total of 415 responses were collected. After preliminary screening, 53 questionnaires with missing data, logical inconsistencies, or careless responses were excluded, resulting in 362 valid questionnaires for data analysis. The demographic characteristics of the participants are presented in Table 1. The gender imbalance observed in our sample (with the number of male participants being approximately three times that of female participants) largely reflects the actual demographic characteristics of the sports lottery gambling population. Prior research has consistently shown that sports betting and gambling activities tend to attract a predominantly male user base, with men participating at substantially higher rates than women. In addition, our recruitment channels (e.g., online sports lottery communities and sports discussion forums) are environments where male users are typically overrepresented, which further contributed to the gender distribution observed in the sample. Therefore, the imbalance is consistent with both the characteristics of the target population and the sampling context.

**Table 1:** Demographic characteristics.

**Variable**	**Category**	***n***	**%**
Sex	Male	275	76.0
	Female	87	24.0
Age	10–19	60	16.6
	20–29	32	8.8
	30–39	189	52.2
	40–49	70	19.3
	Over 50	11	3.0
Total		362	100

### 3.2 Research tools

#### 3.2.1 SRP scale

Drawing on Kim (2017) and related studies, a SRP Scale was developed to assess the level of SRP among Chinese SLGs. The scale consisted of 14 items across four dimensions: economic risk, interpersonal risk, psychological risk, and physical risk. Responses were rated on a five-point Likert scale. Confirmatory factor analysis (CFA) indicated acceptable model fit (CMIN/df = 3.916, CFI = 0.953, RMSEA = 0.090, RMR = 0.045). Reliability analysis showed that Cronbach's α values for the subscales ranged from 0.890 to 0.945, demonstrating good reliability and validity of the scale.

#### 3.2.2 Scale for assessing lottery addiction behavior

This study employed a measurement instrument adapted from the Diagnostic and Statistical Manual of Mental Disorders, Fourth Edition (DSM-IV) of the American Psychiatric Association. This diagnostic tool has been widely used in clinical and empirical research on gambling addiction and has demonstrated strong reliability and validity. Building on this framework, the Gambling Addiction Behavior Questionnaire developed by Jong et al. (2006) was also consulted, and several items were adapted and localized to reflect the context of China's sports lottery.

The scale adopted a single-factor structure and comprised 10 items, each rated on a five-point Likert scale. CFA indicated acceptable model fit (CMIN/df = 4.158, CFI = 0.972, RMSEA = 0.094, RMR = 0.031). Reliability analysis revealed a Cronbach's α coefficient of 0.944, demonstrating strong reliability and validity of the scale.

#### 3.2.3 Scale of SS

Based on Kim (1995) and related studies, a SS Scale was developed to assess the level of SS among Chinese SLGs. The scale consisted of 25 items across four dimensions: emotional support, informational support, instrumental support, and appraisal support. Responses were rated on a five-point Likert scale. CFA indicated acceptable model fit (CMIN/df = 2.493, CFI = 0.958, RMSEA = 0.064, RMR = 0.036). Reliability analysis showed that Cronbach's α values for the subscales ranged from 0.938 to 0.964, demonstrating excellent reliability and validity of the scale.

### 3.3 Data processing and analysis

Data analyses were conducted using SPSS 26.0 and AMOS 25.0. First, reliability analysis, exploratory factor analysis (EFA), and CFA were conducted to examine the reliability and validity of the measurement scales. Second, Pearson correlation analysis was employed to test the relationships among the variables. Finally, hierarchical regression analysis was performed to examine the significance of the moderating effect of SS on the relationship between SRP and gambling addiction. All statistical symbols (e.g., *p, t, F*, η*p*^2^) were formatted in accordance with APA 7th edition guidelines.

## 4 Results

### 4.1 Correlation analysis between variables

As shown in Table 2, SRP was positively correlated with gambling addiction, whereas SS was negatively correlated with gambling addiction. These findings are consistent with the hypothesized relationships among the variables, indicating good theoretical plausibility. Further Pearson correlation analysis revealed coefficients ranging from −0.440 to 0.717, all below the multicollinearity threshold of 0.80 (Hair and Anderson, 2018), suggesting that multicollinearity was not a concern among the variables.

**Table 2:** Scale correlation analysis.

**Variable**	**Economic risk**	**Interpersonal relationship risks**	**Psychological risk**	**Physical risks**	**Emotional Support**	**Information Support**	**Material support**	**Reviews Support**	**Addictive behavior**
1	1								
2	0.596^**^	1							
3	0.521^**^	0.556^**^	1						
4	0.499^**^	0.407^**^	0.448^**^	1					
5	−0.266^**^	−0.114^*^	−0.224^**^	0.066	1				
6	−0.395^**^	−0.329^**^	−0.339^**^	−0.103	0.717^**^	1			
7	−0.246^**^	−0.181^**^	−0.128^*^	−0.110^*^	0.642^**^	0.653^**^	1		
8	−0.245^**^	−0.105^*^	−0.236^**^	0.009	0.715^**^	0.585^**^	0.560^**^	1	
9	0.420^**^	0.393^**^	0.401^**^	0.258^**^	−0.317^**^	−0.440^**^	−0.334^**^	−0.388^**^	1

^*^p < 0.05, ^**^p < 0.01, ^***^p < 0.001.

### 4.2 The relationship between perceived social risk and lottery addiction behavior

Table 3 presents the regression results examining the effects of SRP on gambling addiction among sports lottery participants. The model yielded an adjusted *R*^2^ of 0.512, indicating that the predictors explained 51.2% of the total variance in gambling addiction and reached statistical significance. Among the subdimensions of SRP, economic risk demonstrated a significant positive effect on gambling addiction (β = 0.253). Psychological risk also showed a significant positive association (β = 0.144). In contrast, interpersonal risk and physical risk did not exhibit significant effects on gambling addiction tendencies.

**Table 3:** The influence of SRP on lottery addiction.

**Category**		***B***	***S.E***	**β**	***t***
Addictive behavior	Constant	1.705	0.135		12.668^***^
	Sex	−1.008	0.071	−0.537	−14.146^***^
	Age	−0.159	0.031	−0.203	−5.220^***^
	Economic risks	0.167	0.033	0.253	5.057^***^
	Interpersonal risk	0.087	0.047	0.091	0.068
	Psychological risk	0.152	0.084	0.144	2.888^*^
	Physical risk	0.016	0.043	0.016	0.367
*R^2^* = 0.520, *adjusted-R^2^* = 0.512, *F* = 64.191, *p* < 0.001					

^*^p < 0.05, ^**^p < 0.01, ^***^p < 0.001.

Overall, the findings suggest that economic and psychological dimensions of SRP significantly contribute to the development of gambling addiction among sports lottery participants, whereas interpersonal and physical risk perceptions did not play a significant role in this study.

### 4.3 The moderating effect of SS in the relationship between risk perception and addictive behavior

This study employed multiple regression analysis to examine the relationship between SRP and gambling addiction among sports lottery participants, as well as to test the moderating role of SS. The detailed results are presented in Table 4. Prior to the regression analyses, multicollinearity was assessed using variance inflation factors (VIFs). All VIF values were below 1.50, indicating no multicollinearity concerns and supporting the robustness of the model estimates.

**Table 4:** The moderating effect of SS on the relationship between SRP and addiction behavior.

**Variable**	**Model 1**		**Model 2**		**Model 3**	
	β	***t***	β	***t***	β	***t***
Sex	−0.543	−13.519^***^	−0.492	−12.699^***^	−0.471	−12.161^***^
Age	−0.191	−5.091^***^	−0.172	−4.666^***^	−0.168	−4.627^***^
Social risk	0.413	11.123^***^	0.371	9.855^***^	0.782	6.127^***^
SS			−0.169	−4.260^***^	0.207	1.751
Social risk ^*^SS					−0.491	−3.366^***^
*R^2^*	0.512		0.535		0.550	
*adjusted-R^2^*	0.507		0.530		0.543	
*F*	123.723		18.145		11.332	

^*^p < 0.05, ^**^p < 0.01, ^***^p < 0.001.

In Model 1, only the independent variable—SRP—was entered into the regression. The model explained 51.2% of the variance in gambling addiction (*R*^2^ = 0.512) and was statistically significant (*F* = 123.723, *p* < 0.001). Social risk exhibited a significant positive effect on gambling addiction tendencies (β = 0.413, *p* < 0.001). Model 2 incorporated the moderating variable, SS. The explanatory power increased to 53.5% (*R*^2^ = 0.535; *F* = 18.145, *p* < 0.001), indicating that the inclusion of SS significantly enhanced model performance. In this model, social risk remained a significant positive predictor (β = 0.371, *p* < 0.001), while SS demonstrated a significant negative effect (β = −0.169, *p* < 0.001), suggesting that individuals with higher perceived SS exhibited lower levels of gambling addiction.

Model 3 further included the interaction term between social risk and SS. The explanatory power rose to 55.0% (*R*^2^ = 0.550), accompanied by a significant change in *F* (*F* = 11.332, *p* < 0.001), demonstrating the meaningful contribution of the interaction effect. Social risk continued to show a significant positive effect (β = 0.782, *p* < 0.001), and the interaction term (social risk × SS) exhibited a significant negative effect (β = −0.491, *p* < 0.01), indicating that SS attenuated the positive association between SRP and gambling addiction.

Overall, these findings support the study hypothesis that SS significantly moderates the relationship between SRP and gambling addiction among sports lottery participants. Specifically, higher levels of SS effectively weaken the positive impact of SRP on gambling addiction tendencies.

### 4.4 Additional analysis

To further examine the hypothesized moderating role of SS, additional analyses were conducted using hierarchical regression models. The overall model indicated that SS exhibited a partial moderating effect on the relationship between SRP and gambling addiction.

Specifically, emotional support and appraisal support significantly moderated the effects of economic risk, interpersonal risk, and physical risk on gambling addiction. The interaction terms (economic risk × emotional support, interpersonal risk × appraisal support, and physical risk × appraisal support) were all statistically significant (*p* < 0.05), indicating that higher levels of SS weakened the positive association between perceived risk and gambling addiction.

These results suggest that individuals with strong emotional or appraisal support are less likely to develop gambling addiction when confronted with various social risks.

## 5 Discussion

### 5.1 The mechanism of SRP influencing lottery addiction behavior

Perceived social risk appears to play an important role in shaping the psychological mechanisms underlying gambling involvement among SLGs. Economic instability, in particular, may heighten feelings of financial insecurity, prompting some individuals to regard gambling as a potential means of relieving economic pressure or achieving short-term gains. Such perceptions can reinforce gambling as a maladaptive coping strategy in times of hardship. This interpretation is consistent with literature suggesting that economic downturns and financial stress are often accompanied by increases in gambling-related behaviors (Jeon, 2019).

At the same time, psychological strain and disruptions in interpersonal relationships may further limit opportunities for meaningful social engagement, potentially leading to feelings of isolation or emotional distress. In these contexts, gambling may serve as a temporary escape from anxiety, loneliness, or relational tension, functioning as a form of avoidance coping. Prior studies have likewise noted that individuals under sustained interpersonal or emotional stress may be more prone to engaging in substitute behaviors that offer short-term emotional relief (Kim and Choi, 2019).

Together, these insights highlight that different dimensions of social risk may contribute to gambling involvement through distinct psychological pathways—economic pressures shaping instrumental motivations, and emotional or relational strain shaping avoidance-oriented motivations. These mechanisms underscore the importance of providing targeted social and psychological support to help SLGs cope with social adversities through healthier strategies rather than maladaptive behavioral outlets such as gambling.

Therefore, to prevent and mitigate such problems, it is essential to establish SS systems that provide both economic and psychological assistance. At the same time, designing and implementing stress-reduction programs and educational initiatives that promote healthy values of sport among SLGs are of particular importance. Through such interventions, sports activities can be effectively redirected toward their healthy and positive essence, thereby reducing the risk of their instrumentalization as a vehicle for gambling.

However, unlike the other sub-dimensions, physical risk did not significantly predict gambling addiction after controlling for economic, interpersonal, and psychological risks. Several explanations may account for this finding. First, the measurement of physical risk in this study mainly captured general perceptions of bodily vulnerability rather than concrete health threats directly linked to gambling behavior. Such measurement coverage may limit its predictive power. Second, multicollinearity tests showed moderate correlations between physical risk and other risk dimensions, raising the possibility of suppression effects, whereby the shared variance with psychological or interpersonal risk diminishes its unique explanatory contribution in the regression model. Third, based on Social Cognitive Theory (SCT), behavioral engagement is more strongly driven by affective states and perceived social contingencies than by distal somatic concerns; thus, physical risk may simply be less salient for SLGs when deciding whether to engage in gambling. Finally, considering that SLGs are generally younger and physically active, the relatively low variability (range restriction) in perceived physical risk may further weaken statistical associations. Together, these explanations indicate that physical risk may operate indirectly—via psychological stress or social strain—rather than serving as a direct antecedent of gambling addiction.

Finally, given that individuals differ in their experiences of tension and stress when confronting social risks, it is important to develop tailored stress-coping interventions that account for personal characteristics and contextual factors. Such interventions can help SLGs adopt healthier and more adaptive coping strategies when facing social pressures, thereby preventing the onset of gambling addiction.

### 5.2 The moderating effect of SS in the relationship between risk perception and addictive behavior

Building upon the results showing partial moderation effects of SS (SS), the present findings suggest that emotional and appraisal support play important buffering roles when individuals face increased social risks. These forms of support appear to alleviate psychological strain and reduce the likelihood of adopting maladaptive coping strategies such as gambling addiction. This suggests that in the context of multiple challenges posed by a risk society, SLGs' involvement in gambling is influenced by psychological changes, which may be strengthened or weakened by recognition, praise, criticism, or negative evaluations from others. Specifically, emotional and appraisal support help alleviate the psychological stress associated with social risks, thereby partially suppressing the development of gambling addiction tendencies.

Chinese scholars 张伯明 et al. (2023) similarly found that higher levels of SS partially alleviated anxiety triggered by diminished perceptions of control during the COVID-19 pandemic. Therefore, SS can be regarded as an important external resource for alleviating individual difficulties and psychological stress. Based on the present findings, it can be inferred that individuals with higher levels of SS are less likely to develop gambling addiction when exposed to multiple social risks. This inference is consistent with Kim and Bae (2007), who reported that among high-risk gambling populations, individuals with stronger SS demonstrated relatively lower tendencies toward addiction even when exposed to gambling. Moreover, SS serves as a key moderator of interpersonal risk, providing buffering and protective effects when individuals face difficulties in social interactions. The higher the level of SS, the less susceptible individuals are to gambling addiction when exposed to interpersonal risk.

It should be noted, however, that our findings did not replicate the negative association between physical health problems and gambling addiction reported by Petry (2006). Several factors may explain this divergence. First, prior studies primarily examined clinical gambling disorder populations, in which medical comorbidities are more prevalent and thus more predictive of gambling severity. In contrast, our sample consisted of community-based SLGs with relatively good health, which likely attenuated the role of physical concerns. Second, earlier work has often assessed objective medical conditions, whereas our study measured subjective perceptions of physical risk, which may operate differently. Third, as behavioral addiction research indicates, psychological and social distress typically exert stronger proximal effects on gambling behavior than general somatic concerns.

Therefore, while prior literature suggests that physical health complications can co-occur with gambling disorder, our results imply that perceived physical risk alone does not independently predict gambling addiction among SLGs. Moreover, the sample showed a substantial gender imbalance, with male participants outnumbering female participants by approximately three to one. Although this pattern mirrors the gender composition commonly reported in the sports lottery gambling population, it nevertheless limits the generalizability of the findings to female gamblers or more gender-balanced populations. Future studies should consider adopting targeted sampling strategies to ensure a more balanced gender distribution, which would help verify whether gender moderates the psychological mechanisms examined in this study.

### 5.3 Theoretical and practical implications

From a theoretical perspective, these findings enrich our understanding of gambling addiction by highlighting the critical role of social context. Traditional models of addictive behavior often emphasize individual factors, but our results underscore that SS can buffer the influence of perceived social risks on gambling behavior, in line with the classic stress-buffering hypothesis (Petry and Weiss, 2009). This suggests that individuals with strong support networks are less likely to resort to maladaptive coping (such as gambling) under stress (Petry and Weiss, 2009). Identifying SS as a key moderating factor thus extends existing theories of behavioral addiction by integrating social environmental resources into the explanatory framework.

Practically, our findings offer valuable insights for intervention and prevention programs targeting problem gambling. The results indicate that strengthening an individual's SS network may directly reduce their vulnerability to gambling addiction. For example, incorporating peer-support groups and community engagement initiatives (e.g., Gamblers Anonymous meetings or similar support forums) into prevention programs can provide at-risk gamblers with shared understanding and accountability to abstain (Gomes, 2007). Involving family members in the intervention process (through family therapy or educational workshops) is another crucial strategy, as family support can help monitor behavior and provide emotional encouragement for change. Additionally, community-based resources—such as outreach programs, financial counseling, and recreational alternatives—should be leveraged to address the underlying social stressors (economic strain, isolation) that drive gambling, thereby offering healthier coping outlets. By enhancing social connectedness and support, intervention programs can harness a known protective factor (Petry and Weiss, 2009), directly informing the design of more effective gambling prevention and rehabilitation strategies.

Beyond its theoretical contributions, this study also provides direct implications for the harm-reduction approach to addictive behaviors. Harm-reduction frameworks emphasize minimizing the negative social and psychological consequences of risky behaviors rather than solely focusing on abstinence. The present findings show that perceived social risk heightens vulnerability to gambling addiction, whereas social support serves as a protective factor that weakens this association. These results suggest that enhancing individuals' social support networks—particularly emotional and appraisal support—may function as a harm-reduction strategy by reducing the emotional distress and maladaptive coping tendencies that drive excessive gambling behavior. In this regard, interventions that strengthen community connectedness, promote supportive interpersonal environments, and provide accessible psychosocial resources can help individuals manage social stressors without resorting to harmful gambling behaviors. By identifying specific social–cognitive mechanisms that attenuate addiction risk, this study offers empirical evidence for integrating psychosocial support into comprehensive harm-reduction policies for gambling-related problems.

## 6 Strengths and limitations

This study makes several important contributions. First, it extends the literature by focusing on Chinese SLGs, a population less explored compared with clinically diagnosed gambling addicts. By examining the mechanisms linking SRP, SS, and gambling addiction, the study provides empirical evidence of psychological processes in a real-world, non-clinical group, which enriches theoretical understanding and offers practical relevance. Second, the study highlights the moderating role of SS, especially emotional and appraisal support, in buffering the impact of social risks on addictive behaviors, thereby offering insights for targeted interventions.

However, certain limitations should be acknowledged. The study employed a cross-sectional design, which restricts causal inferences between variables. Longitudinal or experimental research is needed to further validate the causal mechanisms identified. In addition, the data relied on self-reported measures, which may be subject to recall bias and social desirability effects. The sample, although diverse, was geographically limited, potentially affecting the generalizability of the findings. Moreover, the study primarily used quantitative analysis; qualitative research could provide richer insights into the lived experiences and psychological dynamics of gambling addiction.

## 7 Conclusions

This study aimed to investigate the mechanisms linking SRP, SS, and sports gambling addiction among SLGs in the context of emerging social risks. Based on empirical analysis, the following key conclusions were drawn:

First, perceived social risk has a significant impact on sports gambling addiction. The findings suggest that increasing exposure to social risks intensifies economic and psychological stress among sports lottery participants, which in turn heightens their tendency toward gambling addiction. In the context of the COVID-19 pandemic, China's rapid transition toward globalization and digitalization has increased the complexity of relationships among individuals, society, and the state. As new risks continue to emerge, individuals are increasingly exposed to uncertainty and insecurity. These patterns highlight that individuals' lives are shaped by broader social environments and that the nature of social risk is deeply connected to political, cultural, and institutional structures. Therefore, strengthening national risk-management systems is essential for safeguarding individuals' safety and psychological well-being. Given the increasing frequency of global crises, developing interventions that reduce risk perception and prevent pathological gambling is necessary and aligns with core principles of modern crisis management. Public lectures, awareness campaigns, and online courses can help sports lottery participants develop a more rational understanding of social risks and strengthen their psychological resilience. Providing psychological counseling and stress-management programs for high-risk groups may reduce their reliance on gambling as a means of coping with real-life stress.

Second, SS partially moderates the association between perceived social risk and sports gambling addiction. Further analyses indicate that among the dimensions of social support, emotional support and appraisal support partially moderate the association between economic risk and gambling addiction. Moreover, appraisal support also moderates the associations between economic, interpersonal, and physical health risks and gambling addiction. In other words, as a key external resource for alleviating stress and coping with difficulties, higher levels of social support are associated with a reduced likelihood of gambling addiction. Therefore, to effectively mitigate the social risks experienced by sports lottery participants and prevent gambling-related problems, systematic and evidence-based social support interventions are urgently needed. For instance, such interventions should integrate family, peer, community, and professional support to establish a comprehensive system encompassing emotional, appraisal, instrumental, and informational support.

Based on the findings, several directions for future research are suggested: First, future research should conduct in-depth qualitative studies on individuals clinically diagnosed with gambling addiction, in order to better capture the underlying psychological mechanisms and behavioral characteristics, thereby informing the design of more effective intervention strategies. Second, future research should incorporate field investigations and expand the geographic scope and population diversity of samples to enhance the generalizability and robustness of findings.

In summary, this study advances the understanding of how social risk, social support, and gambling behavior are interrelated, and provides a theoretical foundation for the development of targeted prevention and intervention strategies. Future research should further extend these investigations and foster stronger integration between sports participation and mental health promotion, ensuring that physical activity functions as a meaningful contributor to individual well-being. Future harm-reduction efforts should emphasize cultivating supportive psychosocial environments, integrating diverse support resources, and coordinating multi-level interventions to fully realize people-centered approaches to addiction prevention and health promotion.

## Data Availability

The raw data supporting the conclusions of this article will be made available by the authors, without undue reservation.
